# Response surface methodology to optimize the green synthesis process of cefotaxime sodium: Collaborative improvement of yield, crystal form control and environmental benefits

**DOI:** 10.1371/journal.pone.0348350

**Published:** 2026-05-14

**Authors:** Wang Yizhang, Li Jing, Yang Mingxi, Ou Shiqiang, Liu Hao, Xie Peng, Zhu Kaimei

**Affiliations:** 1 Guilin Medical University, Guilin, Guangxi Zhuang Autonomous Region, China; 2 Second Affiliated Hospital of Guilin Medical University, Guilin, Guangxi Zhuang Autonomous Region, China; 3 Guangxi Kelun Pharmaceutical Co., Ltd., Guilin, Guangxi Zhuang Autonomous Region, China; University of Kotli, PAKISTAN

## Abstract

**Objective:**

Based on the response surface method combined with the concept of green chemistry, the synthesis process of cefotaxime sodium (CTX) was optimized, taking into account product yield, quality and environmental benefits, and providing a scientific basis for its industrial production.

**Methods:**

Taking the yield of CTX as the response value, based on single-factor experiments, the Box-Behnken response surface method was used, and the reaction temperature, eluent dosage and salt-forming agent ratio were selected as influencing factors to construct a process optimization model and screen the optimal conditions. The related substances of the optimized product were determined by high-performance liquid chromatography (HPLC), and the crystal form and morphological characteristics of the product were characterized using X-ray powder diffraction (XRD) and scanning electron microscopy (SEM). At the same time, green chemistry evaluation software was used to evaluate the environmental performance of the process.

**Results:**

The response surface regression analysis showed that the optimal process conditions were: reaction temperature 14.8°C, eluent dosage 104.4 mL, and salt-forming agent ratio 1:1. Under these conditions, the yield of CTX was stably achieved at 93.66%; HPLC analysis results showed that the total impurity content of the product was only 3.93%, and the active ingredient content reached 96.07%; XRD and SEM characterization confirmed that the optimized product had a regular crystal form and good crystalline integrity; the green chemistry evaluation score was 94.44, indicating that the process has excellent environmental performance.

**Conclusion:**

The synthesis process of CTX optimized by response surface methodology can not only achieve stable and high product yield, but also ensure product quality that meets stringent control criteria. It also has excellent environmental performance and safety, integrating production efficiency, quality control and green development needs, and has significant industrial application value and promotion prospects.

## Introduction

CTX is a cephalosporin antibiotic first marketed in Germany in 1980. It has the characteristics of broad antibacterial spectrum, excellent chemical stability, and high safety in clinical use. It can effectively treat acute and chronic bacterial infections in the respiratory tract, urinary system, skin, soft tissue and other sites caused by susceptible bacteria, and exerts remarkable clinical efficacy [1–2]. Recent studies have shown that CTX can also be used to treat organ dysfunction diseases such as cirrhosis and sepsis [3–5]. However, in terms of synthesis process, referring to the original patent and existing literature [6–7], there are two key problems in the traditional synthesis process of CTX: 1. The use of highly toxic organic reagents, such as triphenylmethane chloride, etc., which exerts a severe negative impact on the environment and does not meet the requirements of green chemistry [8–9]; 2. Since the reaction-derived impurities are difficult to remove, the quality control is unstable and the yield fluctuates significantly [10]. At the same time, existing research mostly focuses on synthesis methods and routes, with limited investigations on various process parameters in the synthesis process, which is not conducive to experimental design and cost reduction in production processes [11–13]. In addition, there is currently a relative lack of reports on the greenness evaluation software of experimental methods, making it difficult to analyze the greenness of methods, which is inconsistent with the trend of growing environmental awareness in pharmaceutical processes [14–16]. The “Guidelines for the Control of Antibiotic Production Wastewater and Solid Waste” issued by the World Health Organization in September 2024 has explicitly stipulated that green chemistry is a key consideration for process optimization [17–18].

Regarding the above-mentioned drawbacks, this study uses green chemistry as the principle, uses Box-Behnken response surface method to verify the reliability and quantitative correlation of the model through statistics and mathematics, and studies the impact of various indicators on the target product, thereby optimizing the synthesis process of CTX [19–20]. Different from previous studies that only focused on single index optimization and lacked systematic green evaluation, this work for the first time realizes the collaborative improvement of CTX yield, crystal form control and environmental benefits, and innovatively applies MoGSA software for quantitative green assessment of the synthesis process, which fills the research gap of green evaluation in CTX production.In order to avoid the use of toxic reagents, this study abandoned the original synthetic process, but replaced highly toxic organic reagents with low-toxic and easily recyclable green solvents, ensuring the stability of product yield while enhancing environmental sustainability. The quality and yield stability of the optimized product are tested through techniques such as chromatographic analysis, X-ray diffraction, and scanning electron microscopy [21–23]. At the same time, the green chemistry assessment software Modified Green Star Area (MoGSA) was used to evaluate the environmental protection of the method [24–25]. It provided feasible solutions to the problems existing in the synthesis process of CTX, and also provided basis and reference for the green and efficient production of pharmaceutical products, promoting the sustainable development of the pharmaceutical industry.

## 1 Materials

### 1.1 Materials and reagents

Isopropyl alcohol, acetone, chromatographic grade methanol (Sichuan Xilong Science Co., Ltd.); sodium isooctanoate, ethyl acetate, disodium hydrogen phosphate, 85% orthophosphoric acid (Shanghai Aladdin Biochemical Technology Co., Ltd.); sodium acetate (Shanghai McLean Biochemical Technology Co., Ltd.); cefotaxime (AD2311016, Guangxi Kelun Pharmaceutical Co., Ltd., purity ≥ 99.0%); ultrapure water.

### 1.2 Instruments and equipment

One-tenthousandth electronic balance (BSA124S-CW, German SARTORIUS company); blast drying oven (DHG-9075A, Shanghai Yiheng Technology Co., Ltd.); magnetic stirrer (DF-101SA, Shanghai Mani Instrument Equipment Co., Ltd.); Ultrasonic cleaner (KQ-500E, Kunshan Ultrasonic Instrument Co., Ltd.); Constant temperature water bath (HH-S1, Changzhou Huaao Instrument Manufacturing Co, Ltd.); HPLC (Shimadzu LC-20ADXR, Shimadzu Enterprise Management Co., Ltd., Japan); C18 chromatographic column (4.6 mm × 250 mm, 5 µm, SHIMADZU); Powder X-ray diffractometer (Xpert-Pro, Malvern Panalytical Co., the Netherlands); Scanning electron microscope (JSM-7900F, JEOL Co., Japan)

## 2 Methods and results

### 2.1 Synthesis of CTX

Added an appropriate amount of cefotaxime acid, isopropyl alcohol and ultrapure water to a round-bottomed flask, dissolved under stirring at 15℃. Slowly added a salt-forming agent composed of sodium isooctanoate and sodium acetate, adjusted the pH to 6.0 with 85% orthophosphoric acid, added 0.2 g activated carbon for decolorization for 30 min after stabilization, filtered and washed the carbon cake with isopropyl alcohol and ultrapure water. Transferred the filtrate to a round-bottomed flask, raised the temperature to 20℃, added ethyl acetate and stirred for 10 min, slowly added isopropyl alcohol dropwise until the solution turned turbid, and allowed crystal growth for 30 min. After stabilization, the temperature was lowered to 5℃, crystal growth was continued for 1 h, then filtered, and the filter cake was washed with an appropriate amount of isopropyl alcohol, and vacuum-dried at 60℃ for 1 h to obtain the target product CTX.

### 2.2 Single-factor experiments

#### 2.2.1 Reaction temperature.

**Fig 1:**
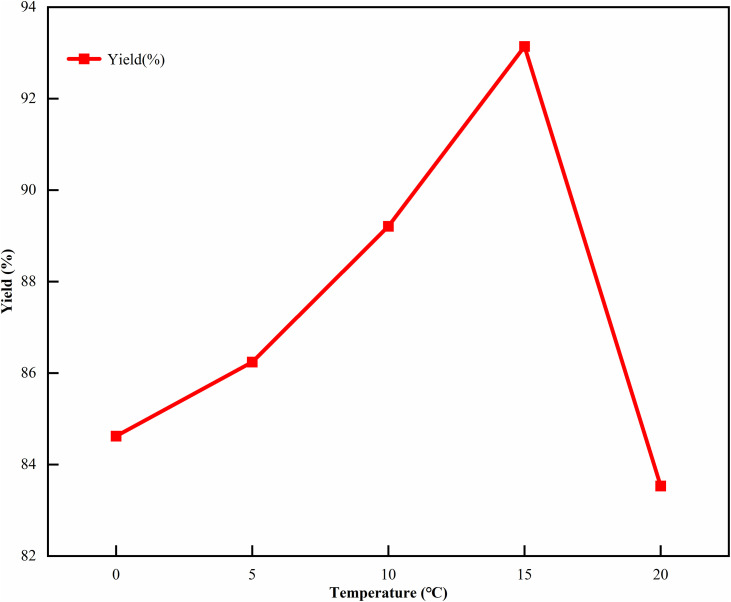
Single factor experimental results of reaction temperature. Here, the reaction temperature was set in the range of 0-20℃, with the dosage of isopropyl alcohol fixed at 100 mL and the salt-forming agent ratio (sodium acetate:sodium isooctanoate) at 1:1 as the controlled variables, and the yield of CTX was the key evaluation index.

Temperature was selected as the investigation factor for the single-factor experiment. This was because when cefotaxime acid reacts with a salt-forming agent to form CTX, the reaction temperature would affect the chemical equilibrium during the reaction process. Elevated temperatures cause hydrolysis of the *β*-lactam ring [26–27], while low temperatures can reduce the reaction rate, so the impact of the 0–20℃ range on the yield needed to be investigated. Fixed the amount of isopropyl alcohol to 100 mL and the salt-forming agent ratio to 1:1. Investigated the effect on the yield when the reaction temperature was 0℃, 5℃, 10℃, 15℃, and 20℃. The results are shown in Fig 1. As the temperature increased, the yield of the target product first increased and then decreased. It reached a peak at 15℃ and the yield exceeded 92%.

#### 2.2.2 The amount of eluent used.

**Fig 2:**
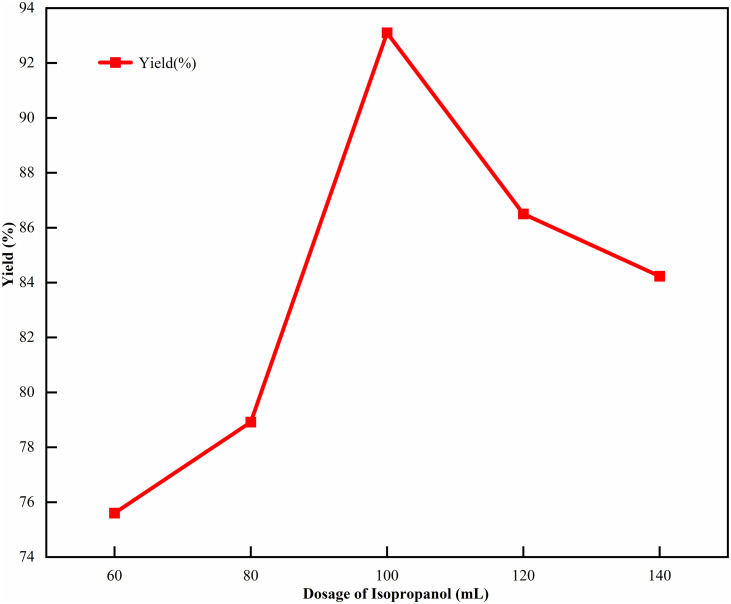
Single factor experimental results of isopropyl alcohol dosage. Here, the isopropyl alcohol dosage was set in the range of 60-140 mL, with the reaction temperature fixed at 15℃ and the salt-forming agent ratio at 1:1 as the controlled variables, and the yield of CTX was the key evaluation index.

The amount of isopropyl alcohol was selected as the variable because isopropyl alcohol, as the eluent, could regulate the polarity of the solution and affect the supersaturation of the crystallization process. Insufficient dosage would lead to incomplete crystallization, while excessive dosage would easily induce impurity formation, so the range of 60 mL-140 mL was examined [28–29]. Fixed the reaction temperature to 15℃ and the salt-forming agent ratio to 1:1. Investigated the impact on the yield when the dosage of isopropyl alcohol was 60 mL, 80 mL, 100 mL, 120 mL, and 140 mL. The results are shown in Fig 2. The variation range of yield in the range of 80 mL-120 mL was larger than other ranges, and when the amount of isopropyl alcohol was 100 mL, the yield exceeded 92%. It showed that adding an appropriate amount of isopropyl alcohol made the crystallization process more stable.

#### 2.2.3 Salt-forming agent ratio.

**Fig 3:**
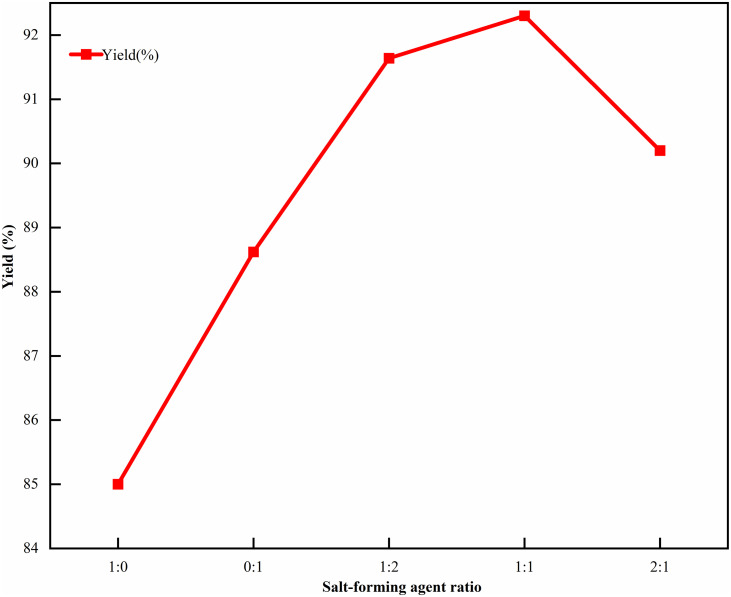
Single factor experimental results of salt-forming agent ratio. Here, the salt-forming agent ratio refers to the molar ratio of sodium acetate to sodium isooctanoate with the range of 1:0-2:1, the reaction temperature was fixed at 15℃ and isopropyl alcohol dosage at 100 mL as the controlled variables, and the yield of CTX was the key evaluation index.

Selected the ratio of salt-forming agent (sodium acetate:sodium isooctanoate) as a variable. Since both salt-forming agents combine with the carboxyl group of CTX by providing sodium ions, the ionic strength of sodium acetate was too high, which can easily lead to local supersaturation, and the ionic strength of sodium isooctanoate was too low, which would cause the crystallization rate to decrease. The ratio of the two may affect the crystallization result, so referred to the 1:0–2:1 range of the previous preliminary experiment [30]. Fixed the reaction temperature to 15℃ and used 100 mL of isopropyl alcohol. Investigated the effect on the yield when the salt-forming agent (sodium acetate:sodium isooctanoate) was 1:0, 0:1, 1:2, 2:1, and 1:1. Since the dosage ratio of the salt-forming agent can regulate the ionic strength of the solution, an appropriate ratio ensures that cefotaxime acid and the salt-forming agent were fully dissolved and contacted, which is conducive to the crystallization of the product from the solution. The results are shown in Fig 3. The yield was the best when the salt-forming agent ratio was 1:1, and then decreased.

### 2.3 Response surface experiment

Based on the single-factor experiment, with reaction temperature, isopropyl alcohol dosage, and salt-forming agent ratio as influencing factors, and yield as the response value, Design Expert 13 software was used to establish a three-factor and three-level method to further optimize the process. The factors and level design were shown in Table 1. The regression equation model between the yield and each factor variable was obtained. Statistical analysis from Design Expert 13 confirmed that a quadratic model was appropriate to describe the relationships. The final quadratic model equation is as follows:*Y* = 93.26–0.3750 *A* + 3.5 *B –* 0.125 *C* + 0.125 *AB* + 0.125 *AC* – 1.62 *BC* – 4.82 *A*^2^ - 8.07 *B*^2^ - 5.32 *C*^2^, The experimental design results were shown in Table 2.

**Table 1:** Response surface factor design.

Level	Temperature (A, ℃)	Dosage of isopropanol (B, mL)	Salt-forming agent ratio (C)
−1	10	80	1:2
0	15	100	1:1
1	20	120	2:1

**Table 2:** Results of response surface experimental design.

No.	A (℃)	B (mL)	C	Yield(%)
1	10	80	1:1	78.0
2	20	80	1:1	77.0
3	10	120	1:1	83.5
4	20	120	1:1	83.0
5	10	100	2:1	83.0
6	20	100	2:1	82.0
7	10	100	1:2	84.0
8	20	100	1:2	83.5
9	15	80	2:1	75.0
10	15	120	2:1	86.5
11	15	80	1:2	76.5
12	15	120	1:2	81.5
13	15	100	1:1	94.0
14	15	100	1:1	92.9
15	15	100	1:1	93.1
16	15	100	1:1	92.3
17	15	100	1:1	94.0

### 2.4 Response surface variance analysis

Analysis of variance for the fitted quadratic model was presented in Table 3. The model was highly significant (*P* < 0.0001) with a non-significant lack of fit (*P* = 0.09 > 0.05), confirming the model’s reliability and predictive validity for process optimization. For all factors, the isopropyl alcohol dosage (B) showed a highly significant main effect (*P* < 0.0001), while reaction temperature (A) and salt-forming agent ratio (C) had non-significant main effects (*P* > 0.05). Notably, the quadratic effects of all three factors (A², B², C²) were highly significant (*P* < 0.0001), and only the interaction between B and C (BC) was significant (*P* = 0.0286 < 0.05); other interactions (AB, AC) were non-significant (*P* > 0.05). The model *P* < 0.05 indicated that the model and experimental results had a good degree of fit. The order of significance of each factor: B(*P* < 0.0001) > A(*P* = 0.3997) > C(*P* = 0.7737), indicating that the amount of isopropyl alcohol had a more significant effect on the yield than the reaction temperature and salt-forming agent ratio. It can be speculated that in the synthesis of CTX, the polarity and supersaturation of the solution had a greater impact on the yield than the reaction rate and ionic strength.

**Table 3:** Variance analysis of response surface.

Source	Sum of Squares	df	Mean Square	*F*-value	*P*-value
Model	654.17	9	72.69	51.93	< 0.0001
A	1.12	1	1.12	0.80	0.40
B	98.00	1	98.00	70.02	< 0.0001
C	0.125	1	0.13	0.09	0.77
AB	0.06	1	0.06	0.04	0.84
AC	0.06	1	0.06	0.04	0.84
BC	10.56	1	10.56	7.55	0.03
A2	97.72	1	97.72	69.82	< 0.0001
B2	274.04	1	274.04	195.80	< 0.0001
C2	119.06	1	119.06	85.07	< 0.0001
Residual	9.80	7	1.40		
Lack of fit	7.62	3	2.54	4.68	0.09
Pure Error	2.17	4	0.54		
Cor Total	663.97	16			

### 2.5 Effect of interaction on yield

**Fig 4:**
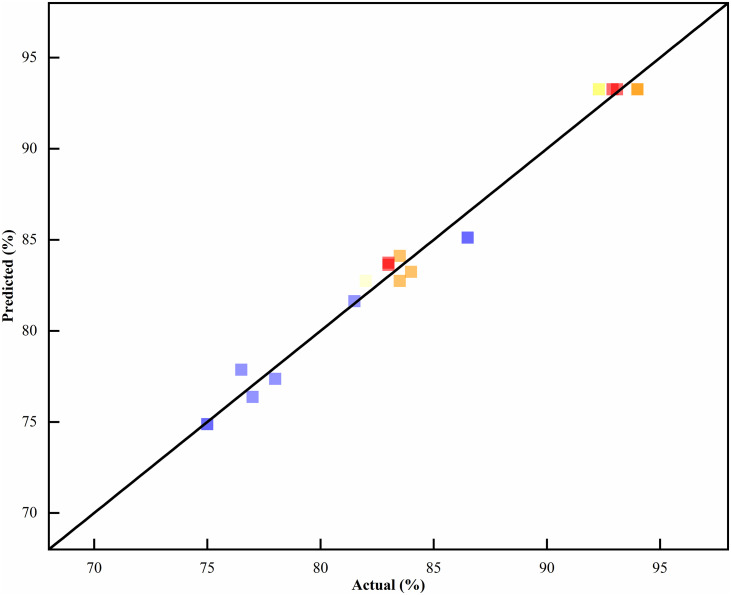
Fitting plot of predicted yield vs. actual yield of CTX synthesis. Here, the abscissa is the experimental actual yield of CTX, the ordinate is the predicted yield of CTX by the response surface methodology model; the solid line is the ideal fitting line, used to verify the model’s predictability.

**Fig 5:**
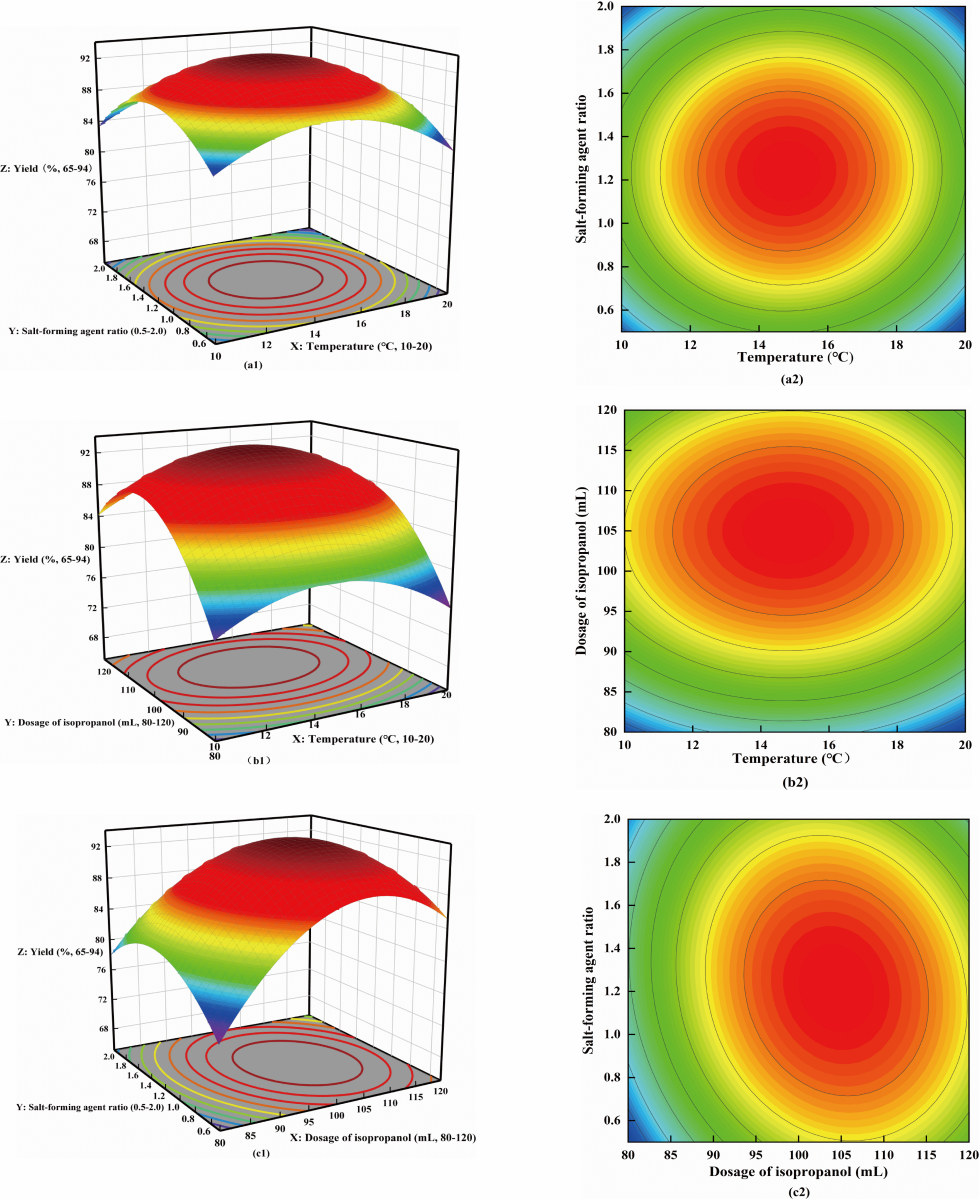
The impact of the interaction of three factors on the yield. Here, (a1) refers to the interaction of reaction temperature and salt-forming agent ratio, (b1) refers to the interaction of reaction temperature and isopropyl alcohol dosage, (c1) refers to the interaction of isopropyl alcohol dosage and salt-forming agent ratio, (a2), (b2), and (c2) show the corresponding optimized contour plots for each interaction. The 3D surface represents the interaction degree of factors, and the contour line represents the influence degree of factors on the yield.

The fitting plot of predicted yield vs. actual yield for CTX synthesis was shown in Fig 4. This plot includes the ideal fitting line, which verifies the good predictability of the response surface methodology model.The interaction diagram between various factors was shown in Fig 5. The 3D response surfaces (Figures 5a1, 5b1, 5c1) represent the degree of interaction between each factor, and the corresponding optimized contour plots (Figures 5a2, 5b2, 5c2) indicate the optimal regions for yield improvement. The dosage of isopropyl alcohol showed a larger curvature in the interaction with the reaction temperature and the proportion of salt-forming agent, indicating that the dosage of isopropyl alcohol had the greatest impact on the yield. It could be seen from the high-line chart that the reaction temperature was slightly larger than the ellipse of the salt-forming agent ratio, and the contours were denser, indicating that the impact on the yield was greater than the salt-forming agent ratio. The conclusion was consistent with the variance analysis results.

### 2.6 Verification of response

Surface optimization results According to the simulation results, the response surface method concluded that the optimal process conditions were a temperature of 14.8℃, a dosage of 104.4 mL of eluent isopropyl alcohol, and a salt-forming agent ratio of 1:1. Three parallel experiments were conducted according to this method, and the product yield results were within the 95% confidence interval, indicating that the response surface model optimization process conditions were reasonable and reliable.

### 2.7 Determination of drug-related substances

#### 2.7.1 HPLC chromatographic conditions.

Mobile phase A: 25.0 mmol·L^-1^ disodium hydrogen phosphate buffer; mobile phase B: methanol; gradient elution: 0−4 min, 5%−20% B; 4−8 min, 20%−40% B; 8−13 min, 40%−20% B; 13−15 min, 20%−5% B; Sample solvent: 5% methanol aqueous solution; Detection wavelength: 235 nm; Injection volume: 10 μL; Column temperature: 35℃; Flow rate: 1.0 mL·min^-1^.

#### 2.7.2 Solution preparation.

Precisely weighed 5.00 mg of CTX reference substance and sample into a 10 mL volumetric flask, used 5% methanol aqueous solution as the solvent, and adjusted to a stock solution with a concentration of 500 μg·mL^-1^. Subsequently, accurately measured 1.00 mL of the stock solution into a 10 mL volumetric flask, and diluted it with the same solvent to prepare a reference substance and test solution with a concentration of 50 μg·mL^-1^.

#### 2.7.3 Preparation of concentration gradient solution.

Precisely measured appropriate amounts of the above test solution, placed them in five 10 mL volumetric flasks, diluted with 5% methanol aqueous solution and adjusted to volume, shook well, and prepared gradient solutions with concentrations of 0.1 μg·mL^-1^, 0.5 μg·mL^-1^, 1.0 μg·mL^-1^, 1.5 μg·mL^-1^, and 2.0 μg·mL^-1^.

### 2.8 HPLC methodology validation

#### 2.8.1 Specificity.

**Fig 6:**
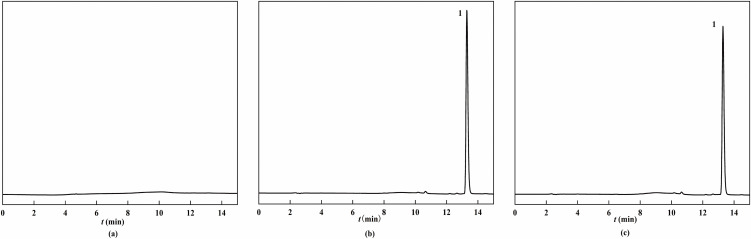
HPLC chromatogram. Here, (a) represents the blank reference solution, (b) represents the CTX reference solution, (c) represents the CTX test solution, and the peak labeled 1 refers to the characteristic peak of CTX with the detection wavelength of 235 nm.

Experiment After the optimized chromatographic conditions were injected, the chromatogram of the test product was shown in Figure 6. The drug peak shape and separation were good, and there was no tailing phenomenon, and the method had good specificity.

#### 2.8.2 Linear experiment.

Injected 10 μL of each of the five gradient concentration solutions, and examined the linear relationship with the peak area as the ordinate (Y) and the concentration of the reference substance as the abscissa (X). The CTX peak area equation was obtained as *Y* = 68968 *X* + 26935(*r* = 0.9992). This shows that the drug has a good linear relationship within the concentration range. The external standard method calculated the CTX content in the test product to be 96.07% and the total drug impurities to be 3.93%.

#### 2.8.3 Recovery rate experiment.

Prepared an appropriate amount of CTX reference solution (50 μg·mL^-1^) as the sample solution. Added the test solution to the reference substances of the above two components to prepare solutions with standard addition amounts of 50%, 100%, and 200% respectively. Each standard addition amount was tested in triplicate to calculate the recovery rate. The average recovery rate of CTX was 98.29%, and the relative standard deviation (RSD) value was 1.98%, indicating that the recovery rate of the method is good.

#### 2.8.4 Detection limit and quantitation.

Limit experiment determined the detection limit and quantitation limit of CTX with signal-to-noise ratios of 3 and 10. The calculated detection limit and quantification limit of CTX were 0.44 ng·mL^-1^ and 1.51 ng·mL^-1^, indicating that the method had good sensitivity and could determine the content of samples with smaller concentrations.

#### 2.8.5 Repeatability experiment.

Took the test solution of the same concentration (50 μg·mL^-1^), injected the sample six times in a row, and measured the RSD value of the peak area. The results showed that the RSD value of CTX was 0.70%, and the repeatability was good.

#### 2.8.6 Stability test.

Took the test solution and stored it in a 3℃ refrigerator for 2 h, 6 h, 10 h, 14 h, 18 h, and 24 h before injecting samples. The calculated CTX peak area RSD value was 1.24%, indicating that the test solution remained stable within 24 h.

### 2.9 X-ray powder diffraction experiment

**Fig 7:**
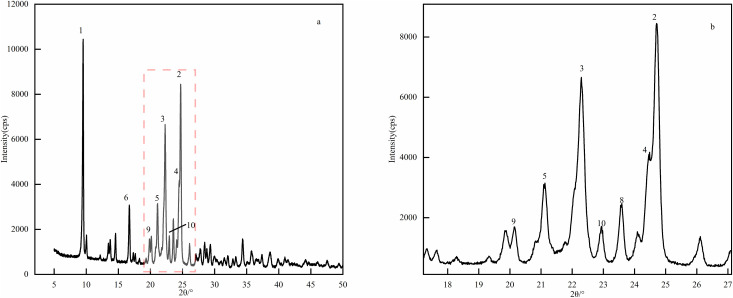
XRD pattern of CTX sample. Here, the XRD test adopted Cu K*α* radiation (λ = 1.541 Å) with the voltage of 45 kV, current of 200 mA, and scanning range of 5-60°, and the half-peak width of the main diffraction peaks was the key evaluation index of crystal integrity.

**Fig 8:**
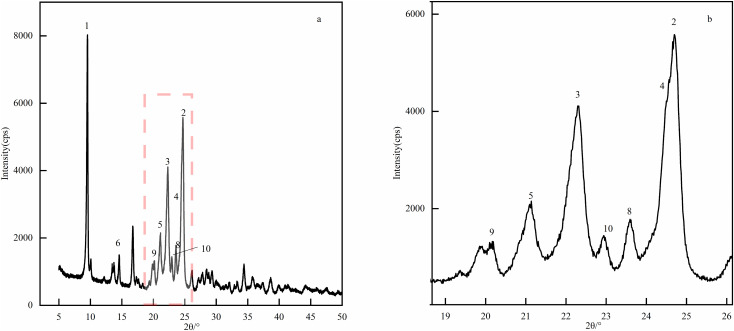
XRD pattern of CTX before optimization. Here, the XRD test parameters were consistent with Fig 7, and the half-peak width of the main diffraction peak was 0.46° as the reference for evaluating the crystal integrity of the unoptimized product.

X-ray diffraction is an efficient non-destructive characterization technology that can accurately identify crystal phases, determine lattice parameters and evaluate sample purity without changing the intrinsic structure and properties of materials. In addition, this technology can also quantitatively analyze key microstructural features such as grain size and residual stress, and is an important characterization technology for drug crystal structures [31,32].The X-ray wavelength was Cu K*α*(λ = 1.541 Å), the voltage was 45 kV, the current was 200 mA, the scanning range was 5–60°, the scanning interval was 0.01°, the scanning rate was 1 s/step, the Soller slit was 5.0°, the divergence slit was 1/2°, and the receiving slit was 20.0 mm. Half-peak width analysis showed that the half-peak width of the second and third main peaks of the optimized sample were 0.23° and 0.29° respectively, which are significantly lower than the half-peak width of 0.46° before optimization, indicating that its crystal integrity was higher(Figs 7, 8).

### 2.10 Scanning electron microscopy

**Fig 9:**
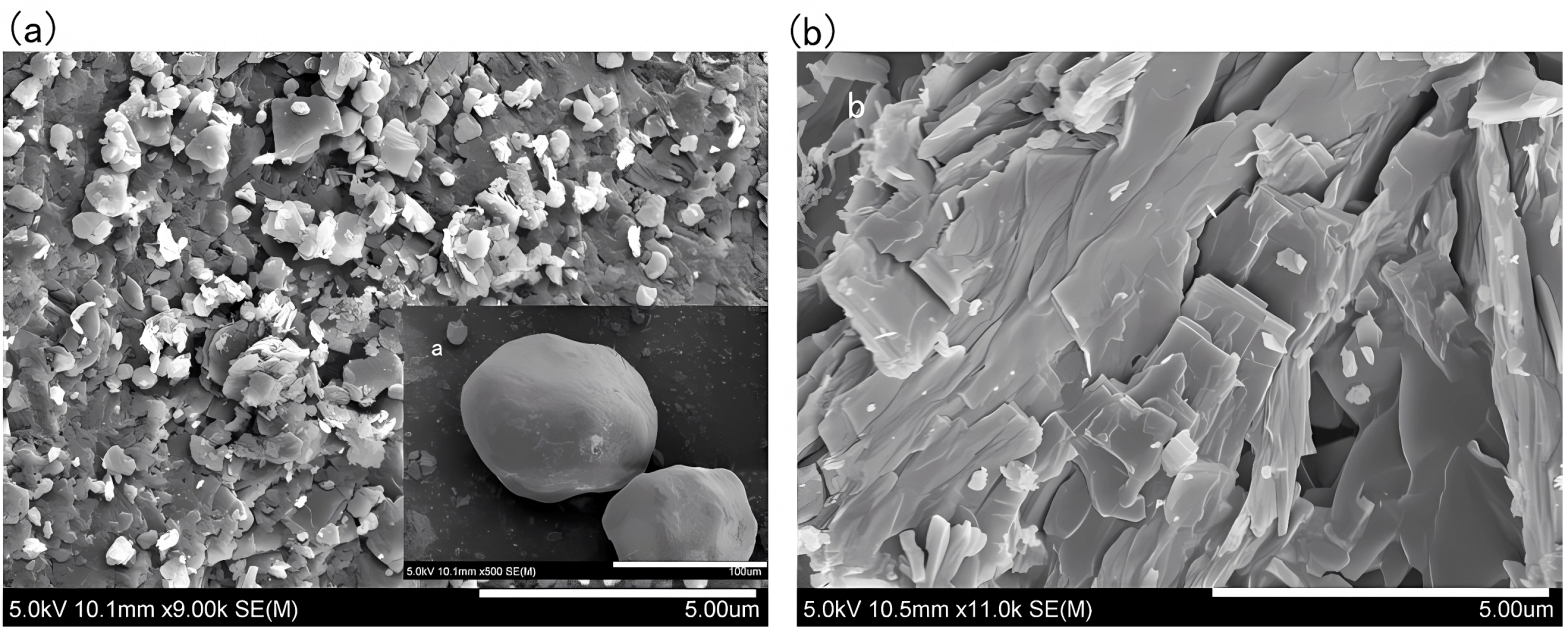
Scanning electron micrographs of CTX. Here, (a) refers to the sample prepared by the traditional synthesis process, (b) refers to the sample prepared by the optimized synthesis process; the SEM test adopted an acceleration voltage of 15 kV, beam current of 1.01 μA, and working distance of 8.9 mm, and the crystal morphology and structure uniformity were the key evaluation indexes.

Scanning electron microscopy is a multifunctional, high-resolution characterization technology that can visually present the surface morphology, microstructure and element distribution of materials with nanometer-level precision. It has good compatibility with solids and powders, and the sample preparation process is relatively simple. It is an important method for characterization of drug crystal forms [33–34].Experimentally, the powder samples were fixed with conductive carbon glue, they were purged with high-purity nitrogen to remove floating powder. Before observation, they were intermittently sprayed with gold under argon protection. A JSM-7900F field emission scanning electron microscope was used, with an acceleration voltage of 15 kV, a beam current of 1.01 μA, and a working distance of 8.9 mm. The secondary electron detector was used for imaging. Fig 9 showed the samples before and after optimization. It could be seen that compared with the sample before optimization, which had a small number of spherical crystals, the sample after optimization had a flaky structure as a whole, and the crystal form is relatively single, which results in better stability of the drug and higher yield.

### 2.11 Green chemistry evaluation

**Fig 10:**
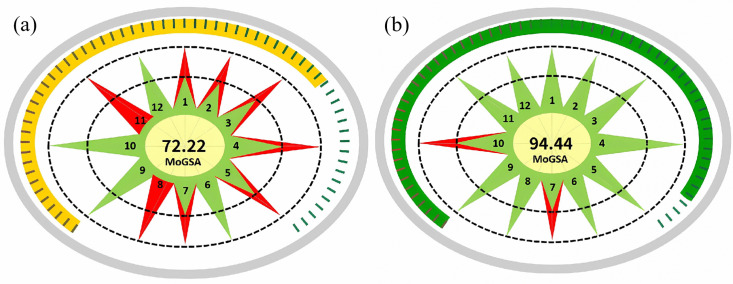
MoGSA results. Here, (a) refers to the original research patented synthesis process, (b) refers to the optimized synthesis process in this study, and the MoGSA score based on 12 green chemistry principles was the key evaluation index of environmental performance.

CTX is a commonly used clinical cephalosporin antibiotic. The original patented process relies on highly toxic solvents such as methylene chloride and N, N-Dimethylacetamide and does not have an environmental protection evaluation system, making it difficult to adapt to modern green production requirements [35]. To evaluate the greenness of the process in this study, we introduced the MoGSA, a quantitative green chemistry evaluation tool developed based on the 12 core principles of green chemistry with a standardized 0–100 scoring scale Weighted scores are assigned to each principle according to its environmental impact intensity, and its evaluation reliability has been validated in the field of pharmaceutical synthesis process. This tool can comprehensively assess the combined impacts of chemical processes on the ecological environment and occupational safety, making up for the limitations of traditional research that only focuses on product yield and quality and ignores environmental performance [36]. MoGSA alone is sufficient for the environmental evaluation of the CTX synthesis process in this study because it fully covers all critical environmental dimensions of CTX synthesis including solvent toxicity, waste generation and energy consumption, and its quantitative scoring system enables direct and objective comparison between the optimized process and the original patented process, which fully meets the greenness assessment needs of this study. Comparing the green characteristics of this process with the original patented process through MoGSA can not only clarify the environmental protection value of process optimization, but also provide data support for the standardization of the green production process of CTX. The evaluation results were shown in Table 4 and Fig 10. The evaluation score was 94.44 points, which was 30.77% higher than the original research process score, indicating that the optimization process has good green environmental protection.

**Table 4:** Evaluation results of MoGSA.

P1-Prevention:	Waste is innocuous
P2-Atom Economy:	Reactions without excess of reagents (= 10%) and without formation of by-products
P3-Less hazardous chemical synthesis:	All substances involved are innocuous
P4 Designing Safer Chemicals:	Chemical products are designed to be fully effective yet have little or no toxicity
P5- Safer solvents and auxiliary substances:	Solvents and auxiliary substances are not used, but if used are innocuous
P6-Increase energy efficiency:	Room temperature and pressure
P7-Use renewable feedstocks:	At least one raw material/feedstock is renewable, water is not considered
P8- Reduce derivatives:	Derivatizations are not used or with one stage
P9-Catalysts:	Catalysts are not used and if used are innocuous
P10-Design for degradation:	All substances involved not degradable may be treated to render them degradable to innocuous products
P11-Real-time analysis for pollution prevention:	Analytical methodologies are in place for real-time monitoring and control to prevent pollution
P12-Safer chemistry for accident prevention:	Substances used with low hazard to cause chemical accidents

## Discussion

In this study, the Box-Behnken response surface method was used to optimize the synthesis process of CTX, and a synergistic improvement in product yield and quality was achieved by constructing a three-factor control model. This study clarifies the quantitative correlation between isopropyl alcohol dosage and CTX crystal form integrity and yield through variance analysis and interaction modeling, which provides a clear parameter control priority for industrial production and makes the process optimization more targeted than previous empirical studies. Response surface variance analysis showed that the model fit was good, and the significance of the influence of each factor on the yield was ranked as isopropyl alcohol dosage > reaction temperature > salt-forming agent ratio, indicating that the impact of solution polarity and supersaturation on the crystallization process was significantly greater than the reaction rate and ionic strength. This result is consistent with the characteristics of the isopropyl alcohol dosage curve in the interaction diagram, which has the largest curvature and the densest contours. It provided a quantitative basis for the priority control of the eluent dosage in industrial production. When the process parameters were optimized to 14.8°C, 104.4 mL of isopropyl alcohol, and 1:1 salt-forming agent ratio, the product yield was stable at 93.66%, and the parallel experimental results fell within the 95% confidence interval, further verifying the reliability and practicality of the model.

The product quality characterization results clearly revealed the synergistic relationship between process, crystal form and quality: XRD analysis showed that the half-peak width of the second and third main peaks of the optimized sample narrowed from 0.46° before optimization to 0.23° and 0.29° respectively, and the crystal integrity was significantly improved. This was attributed to the low temperature of 14.8°C inhibiting the crystal defects caused by the hydrolysis of the *β*-lactam ring, and the precise control of solution supersaturation by 104.4 mL of isopropyl alcohol to reduce disordered crystallization. SEM observation found that the product changed from a small amount of spherical shape before optimization to a regular lamellar crystal form. This is attributed to the 1:1 ratio of the salt-forming agent balancing the ionic strength of the solution, avoiding local supersaturation of sodium acetate or too slow crystallization of sodium isooctanoate, and promoted directional crystal growth. The flaky crystal form not only reduced the specific surface area, but also helped reduce impurity adsorption sites, and also improved filtration and drying efficiency. The experimental results echo the HPLC detection and analysis: the total impurities of the product were only 3.93%, and the content reached 96.07%, and the detection method verification showed that linearity, repeatability, stability, etc. met the requirements of the 2025 version of the Chinese Pharmacopoeia, confirmed the effectiveness of process optimization in quality control [37].From the perspective of industrial feasibility, the optimized process has inherent advantages for potential scale-up: raw materials are low-cost and easily available in bulk, process parameters are clearly defined and controllable, and the green solvent system aligns with industrial environmental policies. Potential scale-up considerations include maintaining uniform temperature in large reactors and stable feeding ratios, which can be addressed through pilot-scale equipment optimization.

In view of the environmental protection points of antibiotic production, this study introduced the MoGSA, a professional green chemistry evaluation software based on the 12 core principles of green chemistry for quantitively assessing the environmental performance and occupational safety of chemical synthesis processes, to realize the environmental protection evaluation of the CTX synthesis process and enhances the completeness and practicality of the research. The optimized process scored 94.44 points, 30.77% higher than the original process, which is attributed to the optimized process fully complying with the key MoGSA evaluation criteria including safer solvents (P5), waste innocuity (P1) and energy efficiency (P6). The core advantage was that it used low-toxic and recyclable isopropyl alcohol and ethyl acetate to replace highly toxic methylene chloride and N, N-Dimethylacetamide, reduced the toxicity of organic waste liquid. At the same time, the reaction temperature was controlled in the range of 0–100°C, without the need for high energy consumption temperature control. It met the requirements of green chemistry priority in the WHO “Guidelines for the Control of Antibiotic Production Wastewater and Solid Waste” [38]. It can not only reduce the impact on the environment, but also help enterprises reduce the economic costs of wastewater treatment, providing environmental protection support for the industrial application of this process. Follow-up research can further expand the screening scope of green solvents, realize dynamic optimization of process parameters based on the actual situation in the drug production process, and promote the development of drug production processes to a higher level of green development.

## Supporting information

S1 File Single-factor experiment data. Includes raw data for plotting the experimental results of reaction temperature, isopropyl alcohol dosage and salt-forming agent ratio.
(XLSX)

S2 File Response surface prediction data. Includes raw data for plotting the response surface prediction results.
(XLSX)

S3 File Response surface interaction data. Includes raw data for plotting the interaction results of experimental factors.
(XLSX)

S4 File HPLC chromatogram data. Includes raw data for the chromatogram analysis of synthesized samples.
(XLSX)

S5 File XRD pattern data. Includes raw data for the diffraction peak analysis of unoptimized and optimized CTX samples.
(XLSX)
