# Correction: Response surface methodology to optimize the green synthesis process of cefotaxime sodium: Collaborative improvement of yield, crystal form control and environmental benefits

**DOI:** 10.1371/journal.pone.0361897

**Published:** 2026-10-30

**Authors:** Wang Yizhang, Li Jing, Yang Mingxi, Ou Shiqiang, Liu Hao, Xie Peng, Zhu Kaimei

The first two authors, Wang Yizhang and Li Jing, should be noted as shared first co-authors on this work.
